# Computational Design of High-Affinity Blockers for Sodium Channel Na_V_1.2 from μ-Conotoxin KIIIA

**DOI:** 10.3390/md20020154

**Published:** 2022-02-21

**Authors:** Guangsi Meng, Serdar Kuyucak

**Affiliations:** School of Physics, University of Sydney, Sydney, NSW 2006, Australia; gmen5776@uni.sydney.edu.au

**Keywords:** sodium channels, conotoxins, molecular dynamics, rational drug design

## Abstract

The voltage-gated sodium channel subtype 1.2 (Na_V_1.2) is instrumental in the initiation of action potentials in the nervous system, making it a natural drug target for neurological diseases. Therefore, there is much pharmacological interest in finding blockers of Na_V_1.2 and improving their affinity and selectivity properties. An extensive family of peptide toxins from cone snails (conotoxins) block Na_V_ channels, thus they provide natural templates for the design of drugs targeting Na_V_ channels. Unfortunately, progress was hampered due to the absence of any Na_V_ structures. The recent determination of cryo-EM structures for Na_V_ channels has finally broken this impasse. Here, we use the Na_V_1.2 structure in complex with μ-conotoxin KIIIA (KIIIA) in computational studies with the aim of improving KIIIA’s affinity and blocking capacity for Na_V_1.2. Only three KIIIA amino acid residues are available for mutation (S5, S6, and S13). After performing molecular modeling and simulations on Na_V_1.2–KIIIA complex, we have identified the S5R, S6D, and S13K mutations as the most promising for additional contacts. We estimate these contacts to boost the affinity of KIIIA for Na_V_1.2 from nanomole to picomole domain. Moreover, the KIIIA[S5R, S6D, S13K] analogue makes contacts with all four channel domains, thus enabling the complete blocking of the channel (KIIIA partially blocks as it has contacts with three domains). The proposed KIIIA analogue, once confirmed experimentally, may lead to novel anti-epileptic drugs.

## 1. Introduction

The voltage-gated sodium (Na_V_) channels are responsible for the initiation and propagation of action potentials, and thus play a key role in membrane excitability and electrical signaling [1,2,3]. The nine subtypes of Na_V_ channel α-subunits (named Na_V_1.1–1.9) are differentially encoded by SCNxA (*x* = 1–5 corresponding to Na_V_1.1–Na_V_1.5, and *x* = 8–11 corresponding to Na_V_1.6–Na_V_1.9, respectively) and expressed throughout tissues [4,5]. Na_V_1.2 was first purified from the rat brain, hence it is also called brain type-II Na_V_ channel. It functions mainly in the central nervous system [6,7]. Genetic mutations in Na_V_1.2 have been linked to seizures, infantile spasm, and autism spectrum disorders. Na_V_1.2, therefore, is studied as a potential target for the development of therapeutics for epilepsy [8].

A class of conotoxins from cone snails (called μ-conotoxins) have been found to target the extracellular vestibule of the Na_V_ channel pore [9] and thus offer promising scaffolds for the rational design of novel peptide-based therapeutic drugs [10,11]. μ-conotoxin KIIIA (KIIIA) contains only 16 amino acids, but retains the typical μ-conotoxins disulfide bond pattern [12], and has shown potential inhibition of TTX-sensitive Na_V_ channels [9,13,14]. KIIIA binds to different Na_V_ channel subtypes with variable degrees of affinity and block, e.g., with 5 nM affinity for rat Na_V_1.2, 37 nM for rat Na_V_1.4, and 97 nM for human Na_V_1.7 [9,14,15]. Experimental structure-activity analysis indicates that the KIIIA residues K7, R10, H12, and R14 are important for binding to various Na_V_ channel subtypes [9,14]. The relative contribution of KIIIA residues in binding to Na_V_ channels differ between the Na_V_ channel subtypes. For example, the neutral replacement of R14 by A14 in KIIIA decreases the affinity for Na_V_1.2 and Na_V_1.4 by two orders of magnitude, whereas the R14A mutation causes only a five-fold reduction in Na_V_1.7. Similarly, the R10A mutation in KIIIA reduces the affinity for Na_V_1.2, Na_V_1.4, and Na_V_1.7, but its effect is much smaller in Na_V_1.7 compared to Na_V_1.2 and Na_V_1.4 [15]. All three channels are incompletely blocked at saturating concentrations of wildtype KIIIA (Na_V_1.2, 90%; Na_V_1.4, 95%; and Na_V_1.7, 94%), which can be compensated by co-binding with tetrodotoxin (TTX) [16].

The recent cryo-EM structure of the Na_V_1.2–KIIIA complex [17] has revealed that KIIIA residues interact with the extracellular segments in domains I to III of Na_V_1.2, and K7 is located near the selectivity filter. The complex structure elucidates the strong polar interaction network at the atomic level. On one side of the toxin, the positively charged side chains of K7 and H12 are coordinated by the negatively charged side chains of E945 and D949, respectively, on the pore helix 2 in domain II. On the other side, the guanidinium group of R10 is coordinated by D1426. In addition, W8 engages in an interaction with Y362 on the extracellular loop of domain I, and the guanidinium group of R14 is H-bonded with the carbonyl group of L920 [17]. The lack of any interactions with any domain IV residues provides an explanation for the partial block of Na_V_1.2 by KIIIA.

Once a toxin is selected as a potential drug lead for a target ion channel, it is important to improve its affinity and selectivity for the target to reduce the dosage and avoid side effects. For toxin peptides, this can be achieved by designing analogues of the toxin through mutations of selected residues, which are predicted to make further contacts with the channel residues. In the case of KIIIA, the residues S5, S6, and S13 are not involved in any interactions with the Na_V_1.2 residues, hence they provide potential mutation sites to improve its affinity for Na_V_1.2.

In this paper, we use computational methods to analyze the molecular mechanism of KIIIA binding to Na_V_1.2. Starting with the cryo-EM structure of Na_V_1.2–KIIIA, we first performed molecular dynamics (MD) simulations to check the robustness of the observed interaction network. Most of the key contacts were found to be preserved during the MD simulations. Then we used the VMD plugin Mutator [18] to generate structures of the KIIIA analogues with the mutations S5R, S6D, and S13K individually and in various combinations. We used the MD simulations to interpret the binding modes of these KIIIA analogues in complex with Na_V_1.2. Compared to the wildtype peptide, we have identified several unique contacts between the KIIIA analogues and Na_V_1.2, which indicate that the mutations could considerably enhance their affinity for Na_V_1.2. Moreover, after the S5R mutation, R5 makes contact with a domain IV residue, resulting in a complete block of the pore. These results suggest that the proposed KIIIA analogues could provide valuable leads in the development of therapeutics for epilepsy.

## 2. Results and Discussion

### 2.1. Critical Interaction Networks Identified from the Na_V_1.2–KIIIA Complex

We start with a comparison of the Na_V_1.2–KIIIA binding mode that was obtained from the MD simulations with that of the cryo-EM structure (Table 1). The average distances between the nearest charged N and O atoms that were computed from the MD simulations were compared to the corresponding experimental values.

Most of the N–O distances that were obtained from MD were in good agreement with the experimental values that are in Table 1, especially when the pair of atoms are at contact distances of ~3 Å when the interaction is strongest. These include the key pairwise interactions, E945–K7, D1426–R10, D949–H12, and L920–R14. A notable difference occurs for the interaction of R14 with E919 and E1444. In the cryo-EM structure, the R14 side chain is in between the side chains of E919 and E1444, and shows a slight preference for the former, whereas in the MD results, the R14 side chain has a distinct preference for the E1444 side chain. 

**Figure 1:**
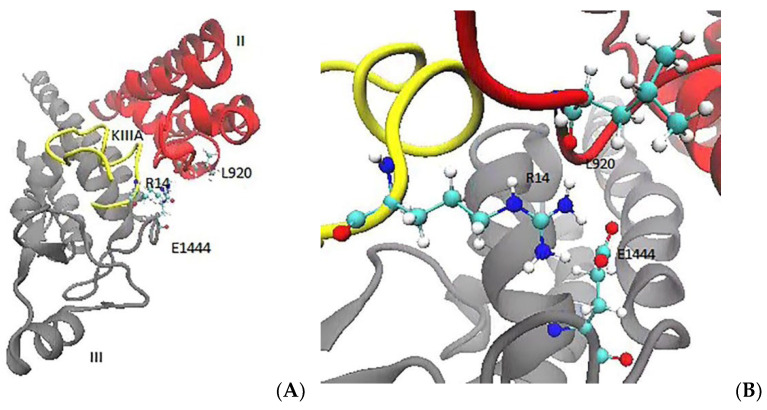
The toxin residue R14 makes contact with the channel residues L920 and E1444 in the MD simulations. (**A**) Extracellular view of R14 depicting its location between the domains II and III. (**B**) A detailed view of R14 interacting with the channel residues L920 and E1444.

R14 is located within the C-terminal tail region, with the side chain extending between the extracellular P1 helix of domain II and the extracellular P2 helix of domain III. The guanidinium group of R14 is wedged between the side chains of E919 and E1444, and it also makes an H-bond with the carbonyl group of L920 (Figure 1). An inspection of the time series of the R14 interactions with L920, E919, and E1444 (Supplementary Materials Figure S1) shows that initially R14 interacts with E919 (which is the bias of the cryo-EM structure), but after a short while it switches to E1444 and forms a stable contact with it. The R14A mutation reduces the KIIIA affinity by two orders of magnitude, which can be explained by the stronger coupling of R14 to the channel residues that were observed in the MD simulations.

### 2.2. MD Simulations of the KIIIA[S5R] Analogue

**Figure 2:**
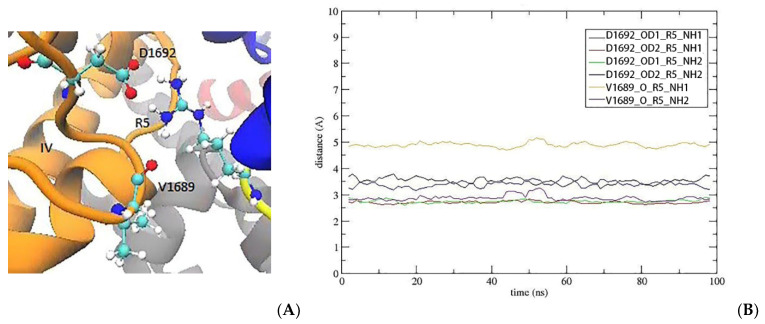
The interacting configuration and dynamic behavior of R5. (**A**) The orientation of the guanidinium group of R5. (**B**) The time series of the R5 interactions with D1692 and V1689.

R5 is positioned near the N-terminal, and its positively charged side chain is in between the channel residues D1692 and V1689 in domain IV (Figure 2A). The guanidinium group of R5 forms a salt bridge with D1692 and makes an H-bond with the main chain carbonyl of V1689. A time series of the pair distances show that R5 makes strong and stable contacts with D1692 and V1689 (Figure 2B), which is expected to make a significant contribution to the binding free energy of KIIIA[S5R].

**Figure 3:**
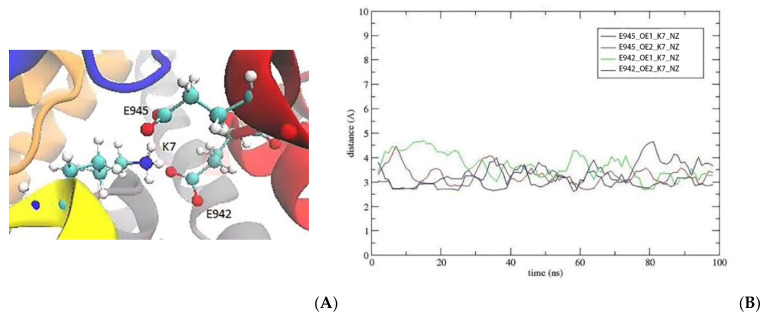
After S5R, the K7 side chain slightly shifts downwards from the EEDD ring towards DEKA, adding E942 contact to the existing E945 contact. (**A**) The side chain orientation of K7. (**B**) The time series of the N–O distances between E942/E945 and K7.

In addition, the S5R mutation introduces a positively charged side chain near the K7 and R10 residues, which perturbs their positions in the Na_V_1.2–KIIIA structure, while other KIIIA residues (W8, D11, H12, and R14) have maintained their contacts after the mutation. The K7 side chain interacts with E945 in the EEDD ring in Na_V_1.2–KIIIA, but after the S5R mutation, it slightly shifts downwards and starts interacting with E942 in the DEKA ring as well, thus gaining extra binding affinity (Figure 3).

**Figure 4:**
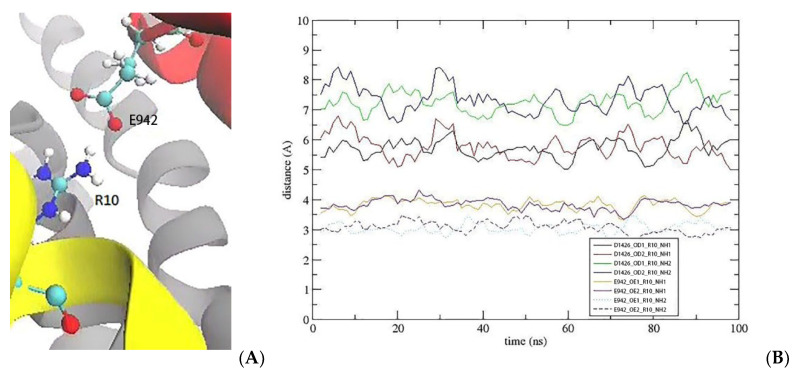
After the S5R mutation, the R10 side chain shifts from D1426 towards E942 in domain II. (**A**) R10 makes contacts with E942 in domain II. (**B**) The pair distances for the E942–R10 contacts.

Similarly, the R10 side chain, which interacts with D1426 in the EEDD ring in Na_V_1.2–KIIIA, shifts towards E942 in the DEKA ring (Figure 4). The R10 side chain is seen to make a more favorable contact with E942 in the Na_V_1.2–KIIIA[S5R] complex compared to its contact with D1426 in the Na_V_1.2–KIIIA complex. Thus, we expect the S5R mutation to indirectly enhance the contribution of the R10 interaction to the affinity.

### 2.3. MD Simulations of the KIIIA[S5R, S13K] Analogue

**Figure 5:**
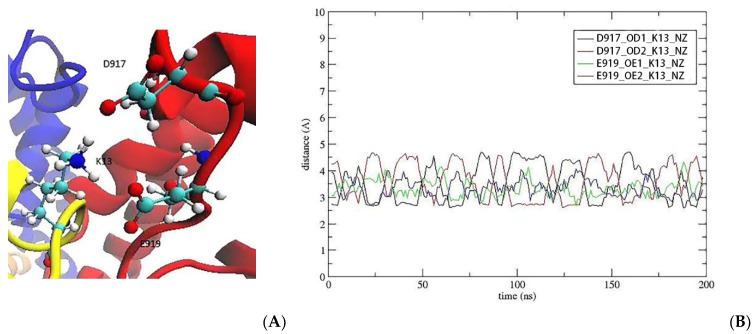
K13 provides a perfect fit for the interaction of K13 with both D917 and E919. (**A**) After the mutation, K13 forms ionic bonds with D917 and E919. (**B**) The time series of the contact distances between K13 and D917/E919.

We next include the S13K mutation in the KIIIA[S5R] analogue. The side chain of K13 is fully extended and forms ionic bonds with the side chains of D917 and E919 (Figure 5A). An inspection of the contact distances in the Na_V_1.2–KIIIA[S5R, S13K] complex shows that K13 makes ionic bonds with D917 and E919 (Figure 5B). Other pairwise interactions have been maintained as in the Na_V_1.2-KIIIA[S5R] complex, so there is no penalty to the binding affinity due to breaking of the existing contacts. Thus, we expect the S13K mutation to increase the affinity of the KIIIA analogue to Na_V_1.2 further.

### 2.4. MD Simulations of the KIIIA[S5R, S6D, S13K] Analogue

**Figure 6:**
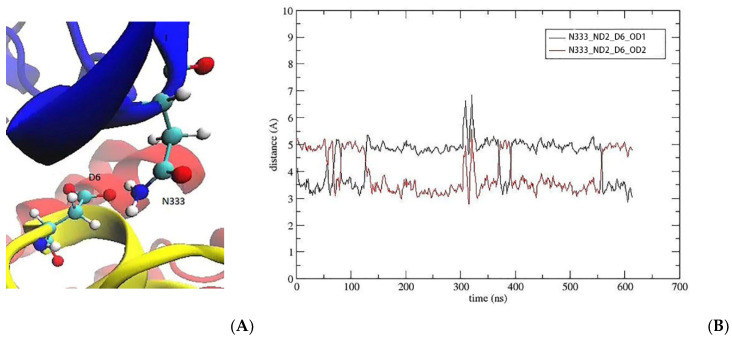
D6 makes an H-bond with N333 in domain I. (**A**) The D6 side chain makes contact with the N333 side chain. (**B**) The time evolution of the D6 contact.

The S6D mutation creates a polar interaction between D6 and N333, which further reinforces the toxin binding in domain I (Figure 6A). The time series of the N–O distances for the D6–N333 interaction indicate a slightly longer contact distance and larger fluctuations compared to those of the R5 and K13 interactions (Figure 6B). Thus, we don’t expect the S6D mutation to contribute to the KIIIA affinity as much as the S5R and S13K mutations. However, for drugs targeting the central nervous system, the peptide absorption is affected by the blood-brain barrier. The S6D mutation introduces one negative charge, which partially neutralizes the surplus positive charges in the KIIIA analogue. After the three mutations, the toxin analogue gains a net charge of +*e*, and the total charge of KIIIA[S5R, S6D, S13K] becomes +5*e*. The binding modes of the Na_V_1.2–KIIIA[S5R, S6D, S13K] and Na_V_1.2–KIIIA complexes are compared in Table 2. It is seen that the pairwise interactions that exist in Na_V_1.2–KIIIA have mostly been maintained after the three mutations which add three more contacts. Therefore, the KIIIA[S5R, S6D, S13K] analogue is expected to yield a substantially higher binding affinity to Na_V_1.2. 

### 2.5. Blocking of Sodium Channel Na_V_1.2

**Figure 7:**
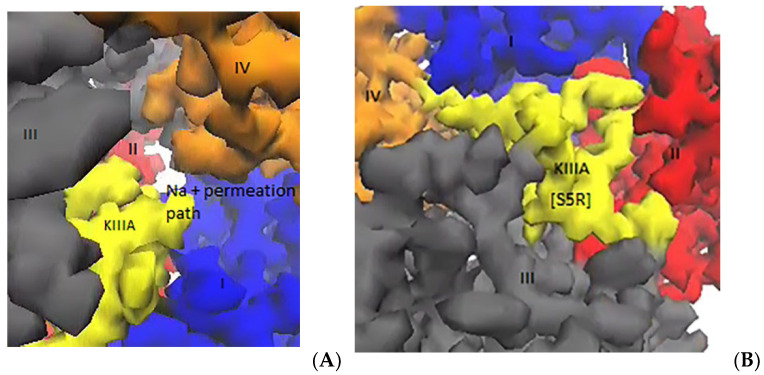
The narrow pathway on the side of domain IV is covered by the new R5 contacts, which results in a complete block. (**A**) There is a partially open ion conduction pathway in domain IV, which allows a sodium ion to get into the selectivity filter. (**B**) The permeation pathway of sodium ions is blocked in the Na_V_1.2–KIIIA[S5R] complex thanks to the interactions of R5 with domain IV residues.

Wildtype KIIIA residues make contact with the extracellular segments in domain I to III of Na_V_1.2, but they make no contacts with domain IV, which leaves a narrow gap on the side of domain IV for entrance of a sodium ion into the selectivity filter. The observation of entry of an Na^+^ ion into the selectivity filter (Figure 7A) is consistent with the incomplete block of Na_V_1.2 with ~95% blockage. After the S5R mutation, the exposed ion permeation pathway is blocked completely by the new contacts between R5 and domain IV residues (Figure 7B), which prevents the permeation of Na^+^ across the toxin analogue. 

## 3. Methods

### 3.1. Modelling of Na_V_1.2 Channel

**Figure 8:**
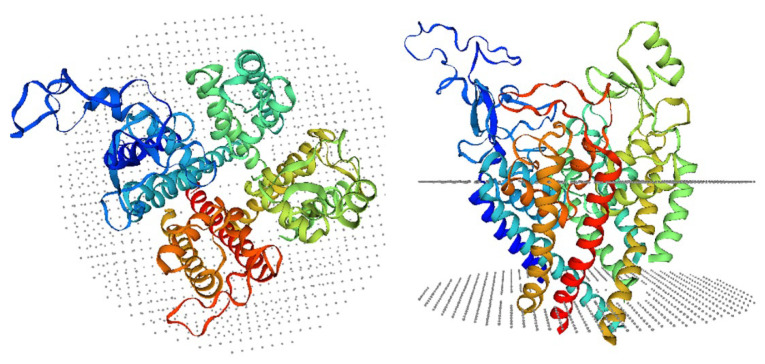
The top view and side view of the pore region of the Na_V_1.2 model that was constructed using SWISS-MODEL. The modelled missing region in domain I is indicated by navy.

We use the cryo-EM structure of human Na_V_1.2–KIIIA (PDB ID: 6J8E) [17] in the MD simulations. As KIIIA binds at the pore domain of Na_V_1.2, we included only the S5–S6 helices and the connecting P-loops and helices in the computations, which correspond to the residues 250–435 in domain I, 884–983 in domain II, 1341–1479 in domain III, and 1655–1780 in domain IV. In the cryo-EM structure of Na_V_1.2, there is a missing piece in the extracellular loop of domain I (residues 285–313) that needs to be included in the structure (Figure 8). The homology model of the Na_V_1.2 pore domain was created with SWISS-MODEL [19]. The sequence and coordinates of the Na_V_1.2 pore domain were submitted as the initial parameters. The target-template alignments were obtained with built-in BLAST [20] and HHblits [21] (Supplementary Materials, Table S1). Based on the alignment, the model was generated using ProMod3 [22], following an energy minimization process (Figure 8).

In order to refine the Na_V_1.2 channel model and check its stability, we performed the MD simulations using the NAMD code [23], employing the protocols that were established in previous MD simulations of Na_V_ channels [24]. Briefly, the channel-toxin complex was embedded in a lipid bilayer and hydrated with a 0.15 M NaCl solution. The simulation system was then relaxed in several steps, gradually relaxing all the restraints on the atoms of the complex system. All the MD simulations were run until equilibration of the system was ensured (typically several hundred nanoseconds).

### 3.2. Search for KIIIA Mutations to Improve Its Affinity for Na_V_1.2

**Figure 9:**
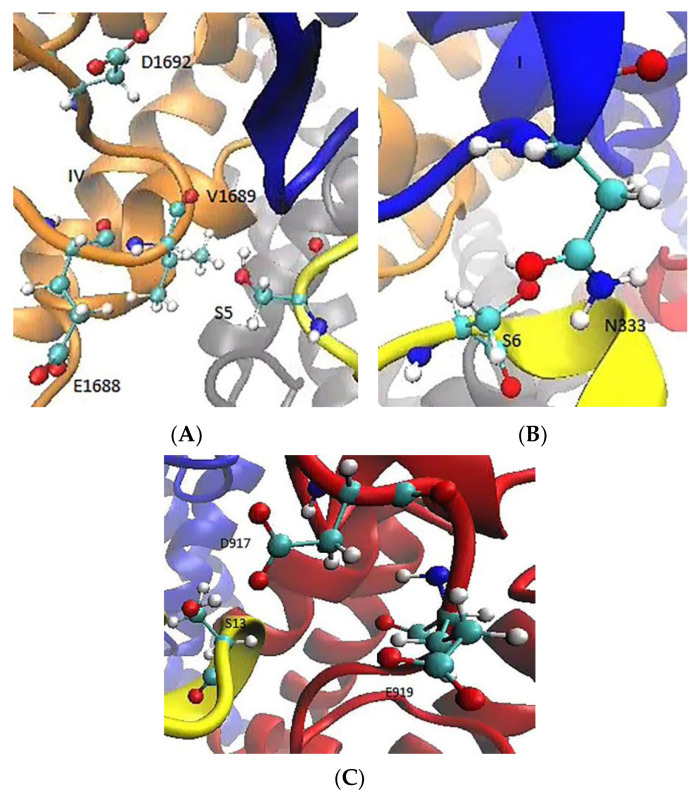
The orientations of S5, S6, and S13 side chains with respect to the channel residues. (**A**) The side chain of S5 is oriented towards E1688 and D1692. (**B**) O_γ_ of S6 is adjacent to N_δ2_ of N333. (**C**) The side chain of S13 points up towards D917 and E919 in domain II.

Excluding the cysteines that are involved in disulfide bonding and those KIIIA residues that already make contact with Na_V_1.2 leaves only S5, S6, and S13 as potential mutation sites to improve KIIIA’s affinity for Na_V_1.2. We inspected the channel residues in the vicinity of S5, S6, and S13 in the Na_V_1.2–KIIIA model, looking for potential charge interactions. We also would like the analogue to achieve a complete block of the channel. S5 is located within the N-terminal region, facing the extracellular loop in domain IV, and pointing towards the residues E1688 and D1692 (Figure 9A). We considered a mutation of S5 to K5 and R5 but ultimately selected R5, which provides more stable contacts with domain IV. For S6, N333 is the only channel residue nearby (Figure 9B), and its mutation to D6 is promising to provide a contact interaction between D6–N333. For the E6 mutation, the side chain is too long to make a good fit with N333. S13 is in the C-terminal region of KIIIA, and its side chain is located between D917 and E919 in domain II (Figure 9C). A mutation of S13 to K13 could provide a perfect fit for the interaction of K13 with both D917 and E919. Therefore, we first considered a mutation of S5R, and then include the S13K and S6D mutations. The Na_V_1.2–KIIIA analogue complexes were constructed from that of Na_V_1.2–KIIIA using the mutator plugin in VMD and refined with the MD simulations.

## 4. Conclusions

In conclusion, we used MD simulations to refine the Na_V_1.2–KIIIA complex and compared the pairwise interactions with that in the cryo-EM structure. The simulations provide a comprehensive understanding of the structural dynamics of KIIIA binding to Na_V_1.2, which are overall in agreement with the reported cryo-EM structure. One feature of the computational modeling are the differences in the R14 binding site. The cryo-EM structure shows that the R14 side chain stably interacts with E919 on the extracellular P1 helix in domain II [17], while our MD simulations indicate that R14 forms a stronger ionic bond with E1444 in domain III. As R14 is identified as a critical residue in KIIIA binding [15], its stronger binding that was observed in our simulations is more consistent with the R14A mutation data.

The Na_V_1.2–KIIIA complex that was constructed here has been used in designing KIIIA analogues with improved binding affinity and complete channel blockage. The S5R mutation forms stable interactions with D1692 in domain IV, which block the ion permeation pathway. The S13K side chain extends towards the extracellular loops in domain II and makes strong ionic bonds with D917 and E919. Both interactions are expected to enhance the binding affinity considerably. However, two positive charges are introduced by the [S5R, S13K] double mutation which may affect the drug absorption. The S6D mutation partly neutralizes the extra positive charges and also its side chain interacts with the adjacent N333 in domain I. The final KIIIA[S5R, S6D, S13K] analogue binds to all four domains of the Na_V_1.2, which results in a complete block of the pore and an increased binding affinity. Our simulation results suggest new potent KIIIA analogues for the blocking of Na_V_1.2. Once tested and confirmed experimentally, they could provide promising leads for anti-epileptic drugs. 

## Figures and Tables

**Table 1:** Comparison of the binding mode of the Na_V_1.2–KIIIA complex that was obtained from the MD simulations with that which was obtained from the cryo-EM structure. The N–O distances in MD were obtained from the average of the last 50 ns of 600 ns MD simulations.

KIIIA		Na_V_1.2		Exp (Å)	MD (Å)
N3	ND2	E330	OE1	2.8	4.4 ± 1.3
			OE2	4.3	5.6 ± 1.0
K7	NZ	E945	OE1	2.5	2.7 ± 0.1
			OE2	4.4	3.1 ± 0.3
W8	NE1	Y362	OH	3.4	3.0 ± 0.2
R10	NH1	D1426	OD1	4.6/5.5	4.8 ± 0.4
			OD2	6.4/5.7	5.1 ± 0.4
	NH2		OD1	2.5/4.0	2.9 ± 0.4
			OD2	4.2/4.4	3.3 ± 0.4
D11	O	R922	N	3.1	3.1 ± 0.2
	OD1	Y1429	OH	2.6	3.0 ± 0.5
	OD2			4.4	4.9 ± 0.4
H12	NE2	D949	OD1	3.1	2.7 ± 0.1
			OD2	3.2	3.1 ± 0.2
		Y362	OH	4.3	3.8 ± 0.4
	ND1	D949	OD1	5.0	4.7 ± 0.1
			OD2	5.0	5.0 ± 0.2
		Y362	OH	5.3	5.4 ± 0.3
	O	N916	N	3.2	4.7 ± 0.4
R14	NH1	L920	O	3.3	3.2 ± 0.7
	NH2			5.4	4.8 ± 0.5
	NH1	E919	OE1	5.0	6.9 ± 1.4
			OE2	5.7	6.7 ± 1.3
	NH2		OE1	4.5	7.5 ± 1.3
			OE2	5.4	7.2 ± 1.2
	NH1	E1444	OE1	6.2	3.8 ± 1.1
			OE2	6.7	3.7 ± 1.2
	NH2		OE1	6.4	3.5 ± 0.6
			OE2	7.3	3.4 ± 0.8
	NH1	Y1443	Center	4.0	5.6 ± 0.7
	NH2			4.6	7.1 ± 0.7
C15	O	M1374	N	3.1	3.0 ± 0.4

**Table 2:** The interacting residues in the Na_V_1.2–KIIIA[S5R, S6D, S13K] complex are compared with those in the Na_V_1.2–KIIIA complex.

Toxin		Nav1.2		WT	S5R, S6D, S13K
R5	NH1	D1692	OD2	/	2.7 ± 0.1
	NH2		OD1	/	2.8 ± 0.2
	NH1	V1689	O	/	4.9 ± 0.2
	NH2			/	2.9 ± 0.3
D6	OD1	N333	ND2	/	3.4 ± 0.4
	OD2			/	4.9 ± 0.2
K7	NZ	E945	OE1	2.7 ± 0.1	2.7 ± 0.1
			OE2	3.1 ± 0.3	4.2 ± 0.6
		E942	OE1	/	2.7 ± 0.2
			OE2	/	4.2 ± 0.4
W8	NE1	Y362	OH	3.0 ± 0.2	3.2 ± 0.4
R10	NH1	D1426/E942	OD1/OE1	4.8 ± 0.4	3.6 ± 0.5
			OD2/OE2	5.1 ± 0.4	3.9 ± 0.3
	NH2		OD1/OE1	2.9 ± 0.4	2.8 ± 0.2
			OD2/OE2	3.3 ± 0.4	3.5 ± 0.4
D11	O	R922	N	3.1 ± 0.2	2.9 ± 0.1
	OD1	Y1429	OH	3.0 ± 0.5	2.7 ± 0.1
	OD2			4.9 ± 0.4	4.0 ± 0.3
H12	NE2	D949	OD1	2.7 ± 0.1	2.8 ± 0.1
			OD2	3.1 ± 0.2	3.0 ± 0.1
		Y362	OH	3.8 ± 0.4	3.5 ± 0.3
K13	NZ	D917	OD1	/	2.7 ± 0.3
			OD2	/	4.5 ± 0.4
		E919	OE1	/	2.7 ± 0.1
			OE2	/	4.0 ± 0.6
R14	NH1	L920	O	3.2 ± 0.7	2.8 ± 0.1
	NH2			4.8 ± 0.5	4.0 ± 0.4
	NH1	E1444	OE1	3.0 ± 0.8	4.7 ± 0.4
			OE2	4.5 ± 0.9	5.7 ± 0.6
	NH2		OE1	3.0 ± 0.4	2.9 ± 0.4
			OE2	3.9 ± 0.7	3.8 ± 0.6
C15	O	M1374	N	3.0 ± 0.4	6.3 ± 0.4

## Data Availability

Trajectory data generated during MD simulations are available from the authors upon request.

## Supplementary Materials

The following supporting information can be downloaded at: https://www.mdpi.com/article/10.3390/md20020154/s1, Table S1: Sequence alignment used in homology modeling, Figure S1: Time series of the R14 contacts with the channel residues.
