# Supplementary material for: Computational Design of High-Affinity Blockers for Sodium Channel NaV1.2 from μ-Conotoxin KIIIA
Source: Mar Drugs. 2022 Feb 21;20(2):154. doi: 10.3390/md20020154 (PMC8880641; doi:10.3390/md20020154)
Supplement: Supplementary file 1 [file marinedrugs-20-00154-s001.zip › marinedrugs-1601888-supplementary.pdf]

Supplementary Materials

Computational Design of High-Affinity Blockers for Sodium Channel NaV1.2 from μ-Conotoxin KIIIA

Sequence alignment used in homology modelling

Table S1. Sequence alignment of the target and uploaded template. The missing residues in the template are indicated by the dashes.

```
The template contained no ligands.

Target                 DVMILTVFCLSVFALIGLQLFMGNLRNKCLQWPPDNSSFEINITSFFNNSLDGNG
template_upload.1.A    DVMILTVFCLSVFALIGLQLFMGNLRNKCLQWPPD--------------------

Target                 TTFNRTVSIFNWDEYIEDKSHFYFLEGQNDALLCGNSSDAGQCPEGYICVKAGRN
template_upload.1.A    ---------FNWDEYIEDKSHFYFLEGQNDALLCGNSSDAGQCPEGYICVKAGRN

Target                 PNYGYTSFDTFSWAFLSLFRLMTQDFWENLYQLTLRAAGKTYMIFFVLVIFLGSF
template_upload.1.A    PNYGYTSFDTFSWAFLSLFRLMTQDFWENLYQLTLRAAGKTYMIFFVLVIFLGSF

Target                 YLINLILAVVAMAYEEQNQATLEEAELTLVLAIIVFIFAVVGMQLFGKSYKECVC
template_upload.1.A    YLINLILAVVAMAYEEQNQATLEEAELTLVLAIIVFIFAVVGMQLFGKSYKECVC

Target                 KISNDCELPRWHMHDFFHSFLIVFRVLCGEWIETMWDCMEVAGQTMCLTVFMMVM
template_upload.1.A    KISNDCELPRWHMHDFFHSFLIVFRVLCGEWIETMWDCMEVAGQTMCLTVFMMVM

Target                 VIGNLVVLNLFLALLLIPSIMNVLLVCLIFWLIFSIMGVNLFAGKFYHCINYTTG
template_upload.1.A    VIGNLVVLNLFLALLLIPSIMNVLLVCLIFWLIFSIMGVNLFAGKFYHCINYTTG

Target                 EMFDVSVVNNYSECKALIESNQTARWKNVKVNFDNVGLGYLSLLQVATFKGWMDI
template_upload.1.A    EMFDVSVVNNYSECKALIESNQTARWKNVKVNFDNVGLGYLSLLQVATFKGWMDI

Target                 MYAAVDSRNVELQPKYEDNLYMYLYFVIFIIFGSFFTLNLFIGVIIDNFNQQKKK
template_upload.1.A    MYAAVDSRNVELQPKYEDNLYMYLYFVIFIIFGSFFTLNLFIGVIIDNFNQQKKK

Target                 IRTLLFALMMSLPALFNIGLLLFLVMFIYAIFGMSNFAYVKREVGIDDMFNFETF
template_upload.1.A    IRTLLFALMMSLPALFNIGLLLFLVMFIYAIFGMSNFAYVKREVGIDDMFNFETF

Target                 GNSMICLFQITTSAGWDGLLAPILNSGPPDCDPDKDHPGSSVKGDCGNPSVGIFF
template_upload.1.A    GNSMICLFQITTSAGWDGLLAPILNSGPPDCDPDKDHPGSSVKGDCGNPSVGIFF

Target                 FVSYIIISFLVVVNMYIAVILENFSVA
template_upload.1.A    FVSYIIISFLVVVNMYIAVILENFSVA
```

Time series of the R14 contacts with the channel residues

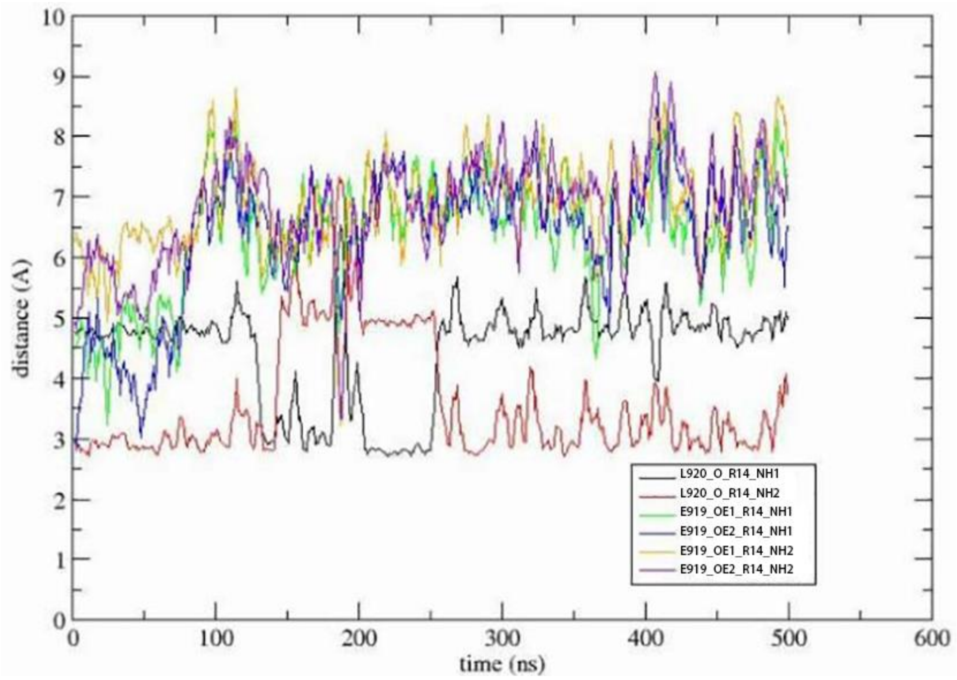

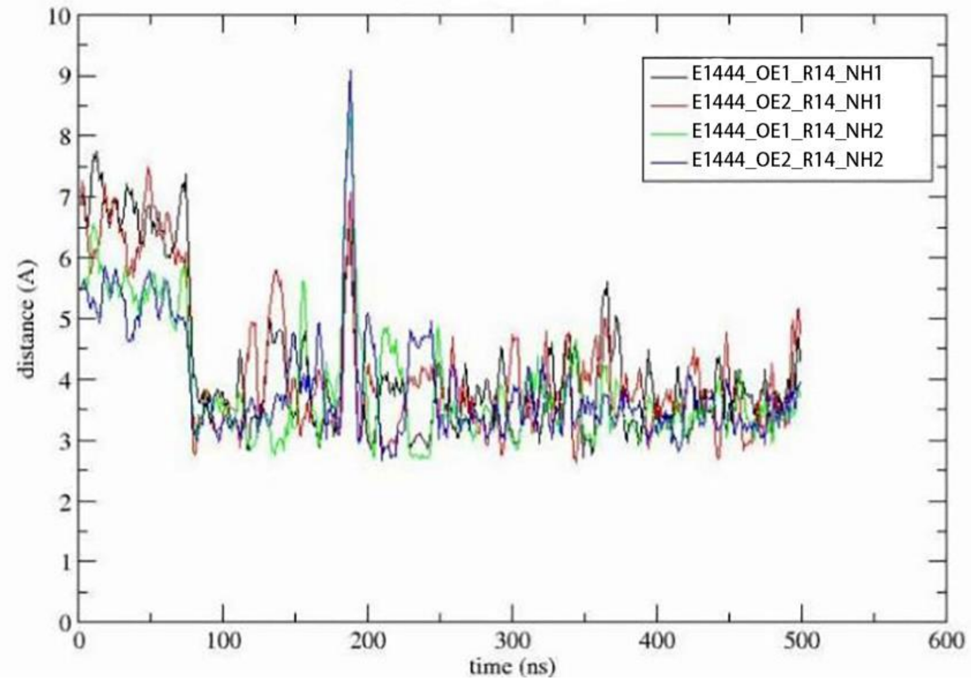

Figure S1. The toxin residue R14 makes contact with the channel residues E919, L920 and E1444. The time series of the E919/L920–R14 and E1444–R14 pair distances in the $Na_V1.2$–KIIIA complex are shown at the top and bottom graphs, respectively. After about 100 ns, R14 is seen to switch from E919 to E1414. The E1444–R14 contact is preserved during the rest of the MD simulations.
